# Critical views for safe surgical phase progression in endoscopic endonasal transsphenoidal pituitary adenoma resection: modified Delphi consensus

**DOI:** 10.1007/s11102-026-01636-2

**Published:** 2026-01-28

**Authors:** Tjasa Zaletel, Danyal Z. Khan, Anjana Wijekoon, Zhehua Mao, Joao Paulo Almeida, Anouk Borg, Jonathan Chainey, Michael D. Cusimano, Daniel A. Donoho, Neil Dorward, Juan Carlos Fernandez-Miranda, Giorgio Fiore, Theofanis Giannis, Alfonso Lagares Gomez-Abascal, Lauren Harris, Abhiney Jain, Ruth Lau, Sacit B. Omay, Igor Paredes, Daniel Prevedello, Gabriel Zada, Danail Stoyanov, Sophia Bano, Hani J. Marcus

**Affiliations:** 1 Department of Clinical and Movement Neurosciences, University College London, London, UK; 2 Department of Neurosurgery, National Hospital for Neurology and Neurosurgery, London, UK; 3 The UCL Hawkes Institute, University College London, London, UK; 4 Department of Computer Science, University College London, London, UK; 5 Department of Neurosurgery, IU Health, Indiana University, Indianapolis, IN USA; 6 Division de neurochirurgie, Département de chirurgie, Université de Montréal, Montréal, Canada; 7 Division of Neurosurgery, Department of Surgery, University of Toronto, Toronto, ON Canada; 8 Division of Neurosurgery, Children’s National Hospital, Washington, DC USA; 9 Department of Neurosurgery, George Washington University School of Medicine & Health Sciences, Washington, DC USA; 10 Stanford University School of Medicine, 213 Quarry Road, Palo Alto, USA; 11 Unit of Neurosurgery, Fondazione IRCCS Ca’ Granda Ospedale Maggiore Policlinico, Milan, Italy; 12 Instituto de Investigación Sanitaria Hospital 12 de Octubre (imas12), Madrid, Spain; 13 Neurosurgery Department, Hospital Universitario 12 de Octubre, Madrid, Spain; 14 Department of Surgery, Faculty of Medicine, Universidad Complutense de Madrid, Madrid, Spain; 15 Department of Neurosurgery, Hospital Universitari Joan XXIII, Universitat Rovira i Virgili, Tarragona, Spain; 16 Department of Neurosurgery, Yale School of Medicine, New Haven, Connecticut USA; 17 Department of Neurosurgery, The Ohio State University and Wexner Medical Centre, Columbus, OH USA; 18 Department of Neurological Surgery, Keck School of Medicine, University of Southern California, Los Angeles, CA USA

**Keywords:** Consensus, CVPP, Delphi, Endoscopic endonasal, Endoscopic transsphenoidal surgery, Pituitary adenoma

## Abstract

**Purpose:**

Endonasal transsphenoidal surgery is the gold-standard for pituitary adenoma resection, yet no intraoperative framework exists to confirm safe phase progression. Inspired by the Critical View of Safety in laparoscopic cholecystectomy and engineering “phase-gate” process, we propose the Critical Views for Phase Progression (CVPPs) – a set of visual cues confirming phase objectives and safe phase progression. Designed to be clinically relevant and machine-readable, CVPPs aim to support training and future AI-driven guidance systems.

**Methods:**

A three-round modified Delphi process was conducted involving 15 pituitary surgery experts from 13 centres across Europe and North America. CVPPs for the naso-sphenoid, sellar, and closure phases were classified as “Essential”, “Desirable” or “Not Necessary”. Consensus required ≥ 70% agreement. A local validation study was subsequently performed involving six experts who reviewed 15 intraoperative video clips and rated their confidence to proceed, which was compared against the predefined reference derived from the finalised CVPPs.

**Results:**

Consensus identified essential and desirable CVPPs across all three phases for both micro- and macroadenoma variants, reflecting differences in exposure goals and surgical risk. Validation demonstrated high concordance between participant ratings and predefined references. Discrepancies arose only in a minority of intentionally incomplete (“unsafe”) views and were attributable to contextual misinterpretation of short video segments, rather than disagreement with the CVPP framework.

**Conclusion:**

This international, multicentre consensus is the first to define CVPPs. By standardising intraoperative visual benchmarks, CVPPs can enhance training, mitigate risks, and provide a foundation for future AI-driven guidance systems capable of real-time anatomical annotation and decision support.

## Introduction

Endoscopic endonasal transsphenoidal surgery has become the gold-standard surgical approach for pituitary adenoma resection, offering a minimally invasive route and reduced morbidity compared to transcranial approaches [1–5]. Despite its widespread adoption, significant variability exists in the performance of the procedure and the resulting outcomes [1, 6–8]. Our group has recently developed a consensus-derived workflow analysis framework for pituitary surgery, which breaks the surgery down into phases (naso-sphenoid, sellar, closure) and their constituent steps, in order to objectively characterise operations [1]. However, the applicability of this framework to improve the quality of surgery intraoperatively in real-time has not yet been realised.

In other fields, workflow analysis has been applied intraoperatively via the Critical View of Safety (CVS) concept [9–12]. This establishes key criteria that must be present to progress safely from one surgical phase to the next. First described by Strasberg in 1995 as a target identification method, CVS in laparoscopic cholecystectomy (LC) promotes the recognition of the cystic duct and artery before proceeding with division to minimise the risk of iatrogenic bile duct injury [13]. It is now considered one of the most important critical factors for overall safety during LC by the Society of American Gastrointestinal and Endoscopic Surgeons expert group [14]. CVS has been used in audit, quality measures, outcome predictions [15–17], and more recently to develop real-time artificial intelligence (AI) models to validate CVS achievement during elective LC [18–21].

**Fig. 1:**
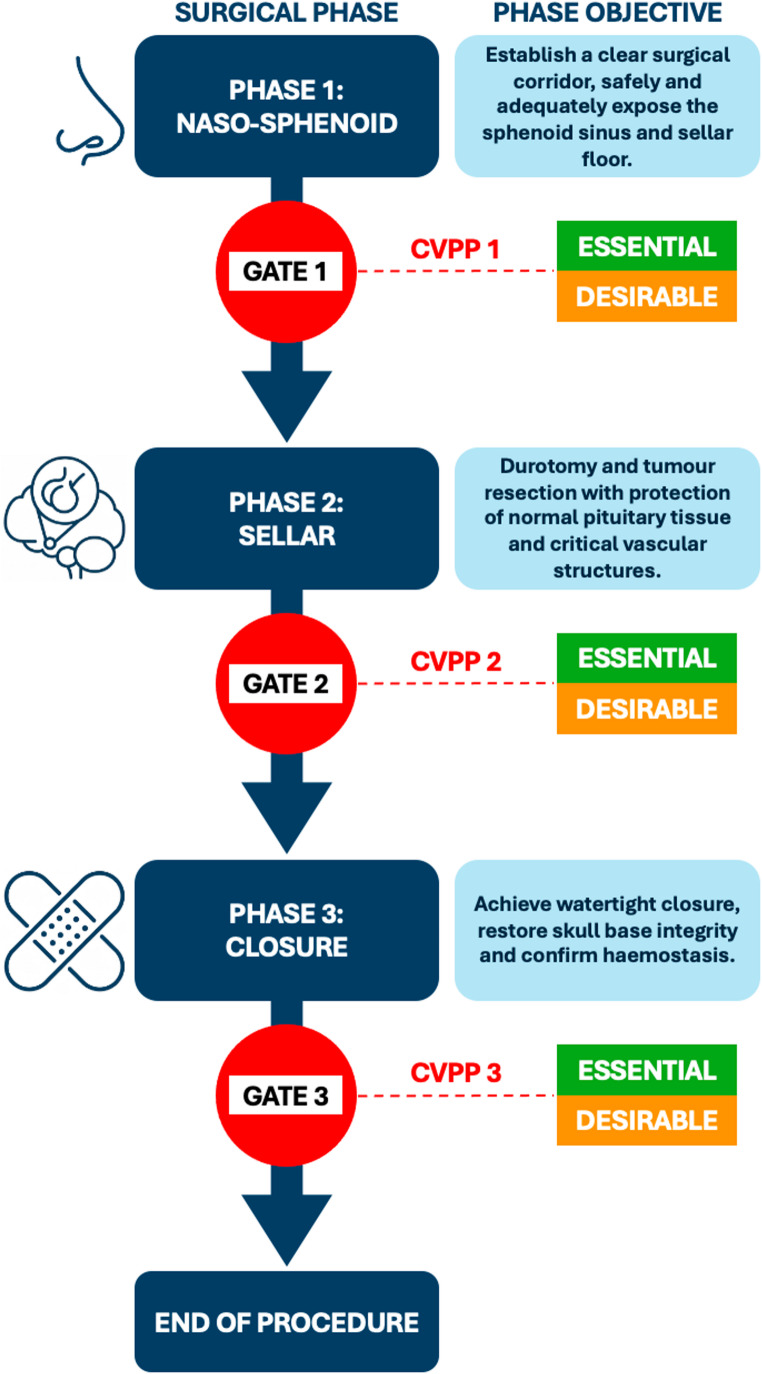
Modified “Phase-Gate” Model for Endoscopic Transsphenoidal Pituitary Adenoma Surgery. Inspired by the Phase-Gate Process (Waterfall Model) used in systems engineering, this modified framework structures the surgical workflow into three distinct phases: Naso-Sphenoid, Sellar, and Closure. Each phase has a clearly defined objective, and progression between phases is governed by three Critical Views for Phase Progression (CVPP 1–3), which act as decision checkpoints (“gates”) requiring confirmation of a set of visual cues before safely progressing to the next surgical phase. These cues are categorised as essential (mandatory for safe transition) and desirable (helpful for enhanced safety or precision but not mandatory), and indicate that the surgical objective of each phase has been successfully achieved, ensuring systematic intraoperative validation, minimising risk, and enhancing surgical standardisation

We sought to adapt a similar approach to pituitary surgery and align it to previous workflow analyses across all surgical phase transitions. We introduce the concept of “Critical Views for Phase Progression (CVPP)”, a set of visual cues that represent completion of the surgical objective of that phase and readiness to proceed to the next phase. While inspired by the CVS concept, CVPPs aim to define mnimum visual criteria for safe phase progression in a complex, multi-phase procedure and, therefore, do not intend to capture full operative nuance. This approach also draws inspiration from engineering principles, particularly the phase-gate process (“waterfall model”), a widely used model in systems engineering and project management to evaluate progress and assess readiness at predefined checkpoints before proceeding to subsequent phases [22–25]. By adapting this method to the surgical workflow, each surgical stage is treated as a distinct phase with a “gate” requiring confirmation of essential visual cues and satisfactory conditions before proceeding (Fig. 1).

This verification process serves multiple purposes. Firstly, it ensures adequate exposure and anatomical orientation, thereby mitigating intraoperative risks (e.g., inadvertent neurovascular injury). Secondly, it enhances surgical standardisation, facilitating surgical training and reducing variability. Finally, beyond its immediate clinical application, it establishes a foundation for future AI-driven intraoperative guidance, as these defined visual cues can serve as training benchmarks for AI systems to assist surgeons in real-time and guide safe progression to the next phase through automated anatomical annotation of the surgical field to improve consistency, efficiency and outcomes.

This study aims to establish an international expert consensus on the visual cues required for safe progression through each surgical phase of endonasal transsphenoidal pituitary adenoma resection and to validate whether these consensus-defined CVPPs reliably capture the visual information needed for safe intraoperative decision-making across different clinical cases and operating surgeons. This is the first such analysis to define phase-specific criteria for all phase transitions within a single operation, providing a standardised framework for intraoperative decision-making.

## Methods

### Overview

This study employed a modified Delphi process [26] to establish consensus on the CVPPs in endonasal transsphenoidal pituitary adenoma resection. These phase-specific visual cues serve as objective intraoperative benchmarks, ensuring that essential procedural goals (e.g., anatomical identification, haemostasis, structural integrity) are met before transitioning to the next phase. Essential cues were defined as mandatory criteria that must be visualised for safe progression, whereas desirable cues are identified as beneficial for surgical precision but not mandatory for phase transition. We aim for these criteria to be both clinically relevant and machine-readable, facilitating their integration into surgical practice and future AI-driven intraoperative guidance systems. For consistency in workflow annotation, the nasal and sphenoid components were combined into a single naso-sphenoid phase to minimise short transitions, simplify workflow segmentation, and facilitate consistency for data structuring and future model training. This study focused exclusively on standard endoscopic transsphenoidal pituitary adenoma resections, excluding transcavernous and other extended endonasal approaches.

### Modified Delphi process and sampling

An initial set of CVPPs and Delphi questions was developed by a local steering committee consisting of three researchers, including a consultant neurosurgeon.

#### Participant selection

A panel of international expert members was selected from our established collaborative pituitary surgery networks based on their experience and expertise in pituitary and skull base surgery, with additional experience in surgical data science (computer vision [CV] and AI). Eligible participants included expert neurosurgeons (i.e., consultants) with active roles in surgical training, research, and guideline development. This ensured that the resulting framework was both clinically grounded and adaptable to future digital applications. Participants were recruited through direct email invitations [27]. Each round included participants from the previous rounds with additional new experts joining as the study expanded. The documents were presented using Microsoft Word (Version 16.94, Microsoft, Washington, USA) and administered using Google Forms (Google LLC, California, USA).

#### Consensus round 1

The initial CVPPs were distributed to a small group of experts (*n* = 9). For each phase, example intraoperative images were provided to illustrate a potential view at the end of that phase, serving as a reference to facilitate the consensus process (Appendix A). Experts were asked a series of questions seeking to assess the accuracy and completeness of visual cues within each CVPP; this included classifying each visual cue as essential, desirable, or not necessary, and providing additional input on missing elements or suggesting modifications for evaluation in subsequent rounds (Appendix B).

According to the Delphi technique, circulation and iterative revision of the visual cues within each CVPP were repeated until data saturation was achieved, i.e., until all experts were satisfied that the CVPPs were complete and accurate. Round 1 was repeated two times, occurring over three weeks (March–April 2025). The final agreement thresholds were pre-defined as ≥ 70% for inclusion and ≥ 85% for exclusion (i.e., rated “not necessary”). Contentious cues (< 70% agreement) were considered in subsequent rounds with clarifying examples and guidance. Any additional suggestions were added if (i) in-scope and (ii) not duplicate.

#### Consensus round 2

Refined CVPPs were then distributed to a larger group (*n* = 11; 9 from prior round, 2 new). Experts were asked to assess the revised CVPPs and expand the defined domains to cover possible global variations in practice. This round was repeated until all the experts agreed that the visual cues reflected the operative practice, and there were no additional suggestions from the participant group. The final inclusion was based on the above-defined consensus thresholds. As with Round 1, additional suggestions were reviewed and added if appropriate. Round 2 was repeated twice, occurring over a three-week period (April–May 2025).

#### Consensus round 3

Round 3 was conducted with an expanded international expert panel (*n* = 15; 11 from previous rounds, 4 new) and focused exclusively on contentious visual cues (i.e., < 70% agreement), along with any new cues proposed by the panel. Visual cues that had already achieved consensus (i.e., ≥ 70% agreement) in prior rounds were fixed and excluded from re-rating. A summary of results from previous rounds was shared with the panel, clearly indicating which visual cues had already reached consensus and which required re-rating. In addition, clarifying examples were added to cues with a high degree of variability to guide consistent interpretation. Round 3 was completed over an eight-week period (July–September 2025), finalising consensus on all remaining contentious visual cues.

### Data collection and analysis

Participant demographics collected included training grade, years of experience in transsphenoidal surgery, and associated institution. The Delphi surveys regarding the CVPPs consisted of Likert-scale items (essential, desirable, not necessary) along with open-ended fields for qualitative input (additional suggestions or comments). Content analysis was used to analyse free-text responses, which included removing out-of-scope suggestions, grouping similar suggestions together, and comparing them to existing data points.

### Validation study

A local validation study was conducted to evaluate whether the finalised CVPPs reliably capture the visual information for safe intraoperative decision-making. The validation was performed using a structured survey (Qualtrics XM, Utah, USA) administered via email to a network of neurosurgeons performing endoscopic pituitary surgery (*n* = 6) at a tertiary neurosurgical centre in the UK, including board-certified surgeons (*n* = 3), those undertaking a dedicated pituitary fellowship (*n* = 2), and a resident with interest in skull base surgery (*n* = 1). The inclusion of surgeons at various stages of training and independent practice was intentional, ensuring that the validation reflected those most likely to utilise and benefit from the CVPP framework in real-world practice.

Participants were shown a total of 15 short (~ 10 s) intraoperative video clips, representing potential endoscopic views at the end of each surgical phase. Clips were chosen instead of static frames to allow assessment of dynamic cues (e.g., CSF leakage, arachnoid movement), and were intentionally short to isolate end-phase visual information and minimise reliance on procedural context. For each clip, experts rated their confidence to proceed using three categories (“clearly safe”, “just adequate – proceed with caution”, “not safe”). Prior to assessment, participants were provided with the finalised CVPP framework and a brief guidance on its intended use. The responses were automatically compared against a predefined reference classification set by our study group; where discrepancies occurred, participants were prompted to provide their reasoning and feedback. Clips were purposively selected to represent both complete (“safe”) and incomplete (“unsafe”) end-phase views. As a guide, our study group classified views missing any essential visual cue as “unsafe”, those containing all essential cues as “just adequate”, and those showing all essential plus at least one desirable cue as “clearly safe” to proceed. For bilateral structures (e.g., carotid prominences), inadequate visualisation of either side was treated as failure to meet the essential criterion. For analysis, ratings were collapsed into a binary outcome (i.e., “safe to proceed” vs. “do not proceed” i.e., combining “clearly safe” and “just adequate” categories) to better reflect real-wold decision-making. Responses were analysed for concordance to assess the reliability and face validity of the CVPP framework for real-world intraoperative application.

### Ethics

This study involved no patient data, participation was voluntary and all participant responses were fully anonymised. All video clips were sourced from our institutional research repository of routinely collected operative recordings, with prior patient consent for research use. The topic was non-sensitive, and data collection did not involve human subjects or clinical interventions; therefore, formal ethical approval was not required in accordance with our local institutional policy (see UCL exemptions).

## Results

### General

Response rate remained high across Delphi rounds (89% in Round 1, 100% in Round 2, 87% in Round 3) with no significant attrition across Delphi rounds. Overall, 15 expert panel members from 13 centres across five countries participated: UK (*n* = 3), USA (*n* = 6), Canada (*n* = 2), Spain (*n* = 3), and Italy (*n* = 1).

### Final CVPP framework

Consensus on CVPPs was progressively refined across three iterative rounds through quantitative rating, structured feedback, and re-assessment of contentious items. Round 1 generated predominantly qualitative input, leading to structural refinements with subdivision of naso-sphenoid and sellar phases into macro- and microadenoma cases to reflect differing exposure objectives between tumour types. Subsequent rounds focused on resolving variability by standardising definitions, providing additional guidance, and clarifying examples. The final set of consensus-defined CVPPs is presented in Table 1.

### Naso-sphenoid phase

Following feedback from Round 1 on exposure sufficiency, the naso-sphenoid phase was subdivided into macro- and microadenoma tumour types to account for differences in surgical objectives. The view at the end of naso-sphenoid phase comprised 7 visual cues for macroadenomas (4 essential, 3 desirable) and 7 for microadenomas (5 essential, 2 desirable), focusing on midline orientation and critical neurovascular landmarks.

Nearly all visual cues in this phase reached consensus. Of note, optic nerve prominences (ONPs) in macroadenomas, and the clival recess and tuberculum sellae in microadenomas, fell just below the predefined consensus threshold, with responses split between “Essential” and “Desirable” categories. Following internal discussion within the study group, these cues were classified in alignment with the classifications used in the alternate tumour subtype to maintain cross-phase consistency and support logical continuity for future model development. Therefore, ONPs were categorised as “Desirable”, and clival recess and tuberculum sellae as “Essential” for both tumour types. For the cavernous sinus, inter-rater variability was resolved when the panel agreed that visualising its margins holds particular importance in invasive microadenomas extending laterally into the medial compartment, leading to consensus.

### Sellar phase

The sellar phase generated the most detailed discussion and greatest inter-rater variability, particularly concerning the degree of tumour resection required before progressing to closure. The final CVPPs were defined separately for macro- and microadenomas, with consensus achieved on 6 visual cues (5 essential, 1 desirable) for each. A small number of cues (i.e., normal pituitary tissue and CSF leak ± arachnoid tear detection in macroadenomas) initially fell below the inclusion threshold with the ratings split between “Essential” and “Desirable” categories. After internal group discussion, both were included in the “Essential” category to maintain consistency between tumour subtypes and to reflect their clinical importance in confirming adequate decompression and haemostasis.

**Table 1:** CVPPs across surgical phases in endoscopic transsphenoidal pituitary adenoma resection

Phase	Tumour type	Visual cues	
		Essential	Desirable
*Naso-Sphenoid*	*Macroadenoma*	• Sellar bulge • Carotid protuberances • Clival recess • Tuberculum sellae	• Opticocarotid recess • Optic nerve prominences • Planum sphenoidale
	*Microadenoma*	• Sellar bulge • Carotid protuberances • Clival recess • Tuberculum sellae • Cavernous sinus margins *(if applicable)*	• Opticocarotid recess • Optic nerve prominences
*Sellar*	*Macroadenoma*	• Sellar fossa clearance of tumour • Sellar floor* visualisation & clearance • Arachnoid dome descent and suprasellar compartment clearance • Residual tumour identification *(if goal debulking*,* then acceptable presence; if goal resection*,* then absence)* • CSF leak ± arachnoid tear detection	• Normal pituitary tissue identification
	*Microadenoma*	• Sellar fossa clearance of tumour • Sellar floor* visualisation & clearance • Residual tumour identification *(if goal debulking*,* then acceptable presence; if goal resection*,* then absence)* • CSF leak ± arachnoid tear detection • Normal pituitary tissue identification	• Medial wall of cavernous sinus inspection
*Closure*	*Macroadenoma* *&* *Microadenoma*	• Haemorrhage control • No active CSF leak • Stability of repair construct • Vascularised flap viability *(if applicable)*	• Repair construct pulsatility *(if applicable)*

* This refers to the surgical floor at the inferior aspect of the resection cavity, which approximately represents the posterior wall of the sella extending to the dorsum sellae anatomically

### Closure phase

Consensus for the closure phase was achieved rapidly with a low degree of inter-rater variability. The CVPPs for this phase were the same for macro- and microadenomas, comprising 5 visual cues (4 essential, 1 desirable) focusing on adequate haemostasis, watertight closure, and construct stability. Early variability regarding flap viability was resolved by clarifying that this cue applies when a vascularised flap is used in reconstruction, achieving full consensus in the final round.

**Fig. 2:**
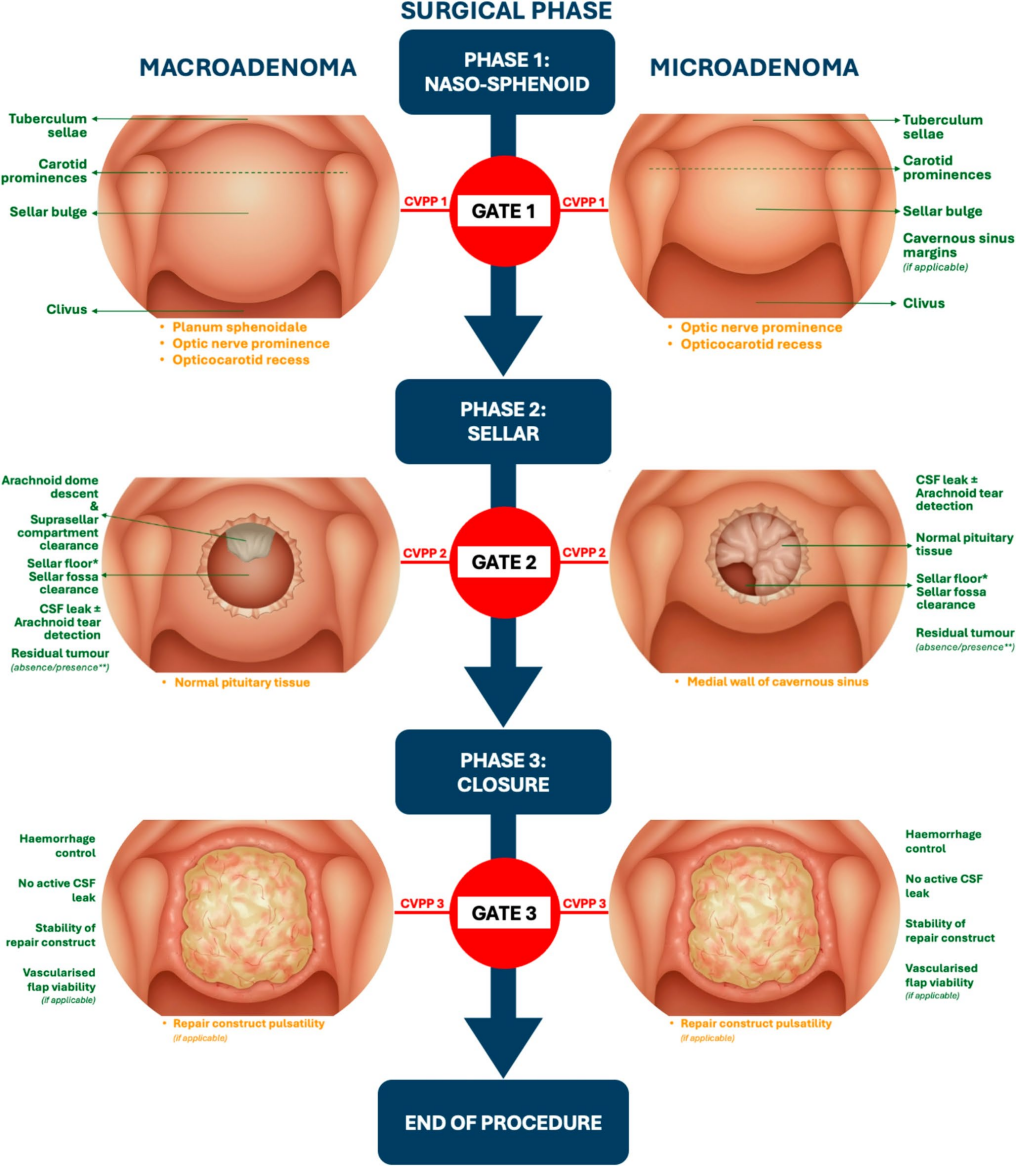

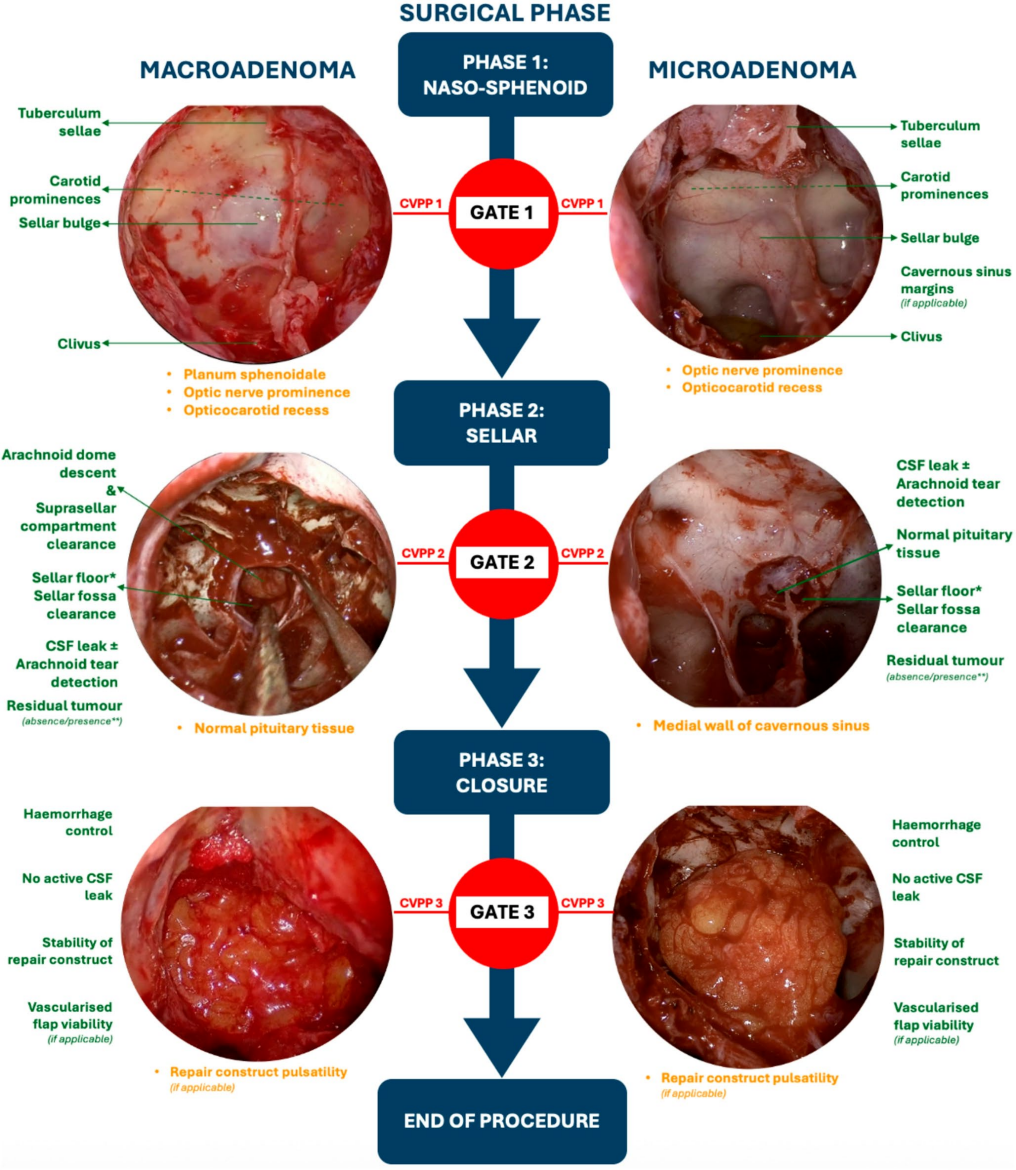
Modified “Phase-Gate” Model with Visual Cues for Each CVPP in Endoscopic Transsphenoidal Pituitary Adenoma Surgery. The diagrams with illustrated (A) and intraoperative endoscopic (B) example views summarise essential (green) and desirable (yellow) visual cues defining safe transition between the naso-sphenoid, sellar, and closure phases as defined by the modified Delphi consensus process. Separate depictions for macroadenoma and microadenoma reflect phase-specific exposure objectives. * This refers to the surgical floor at the inferior aspect of the resection cavity, which approximately represents the posterior wall of the sella extending to the dorsum sellae anatomically. ** If goal debulking, then acceptable presence; if goal resection, then absence. **A** Schematic illustration of the modified phase-gate CVPP framework. **B** Annotated intraoperative endoscopic example views of CVPPs

The final set of CVPPs represent a unified international consensus on essential and desirable visual cues for safe phase progression in endoscopic pituitary adenoma resection. Figure 2 illustrates their integration within the modified phase-gate framework, showing (A) schematic depictions alongside (B) real-life annotated intraoperative examples that demonstrate how these critical views appear under operative conditions.

### Validation study

A total of 90 responses were collected, which were compared to our predefined reference classification. A summary of participant agreement and qualitative feedback in cases of discordance is presented in Table 2.

**Table 2:** Validation study results - participant agreement with reference classification with summary of feedback for discordant cases

PHASE	TUMOUR TYPE	No. of clips	Reference classification	Agreement with reference (%)	False-proceed cases (*n*, %)	False-hold cases (*n*, %)	Qualitative feedback for discordance
*Naso-sphenoid*	*Macro*	3	2 safe; 1 unsafe	94%	0	1 (8%)	Limited carotid visualisation
	*Micro*	3	2 safe; 1 unsafe	89%	2 (33%)	0	Adequate exposure with endoscope repositioning
*Sellar*	*Macro*	3	2 safe; 1 unsafe	100%	0	0	
	*Micro*	3	2 safe; 1 unsafe	89%	2 (33%)	0	One participant acknowledged misjudgement; another noted further resection is required
*Closure*	*Macro & Micro*	3	2 safe; 1 unsafe	100%	0	0	

Participant ratings showed high concordance with the predefined reference, with a mean agreement of 94% across all clips. Agreement was complete for all but one “safe” clip (i.e., 9 out of 10), confirming that the CVPPs accurately reflected intraoperative readiness to proceed. The single instance of disagreement occurred when one participant rated a view as unsafe due to perceived incomplete visualisation of the carotid protuberances and requested additional navigation data, reflecting caution rather than disagreement with the CVPPs.

Across all subphases, five clips represented intentionally “unsafe to proceed” views with lower agreement levels. Two instances of partial discordance were noted, with 2 out of 6 participants (33%) rating the view as “safe”; both involved microadenoma cases with incomplete carotid (naso-sphenoid phase) or sellar floor visualisation (sellar phase). Qualitative feedback suggested these discrepancies reflected uncertainty about surgical stage – specifically, whether clips represented exposure or resection endpoints, rather than the CVPP criteria.

## Discussion

### Principal findings

This study is the first to define “Critical Views for Phase Progression (CVPPs)” and apply them in the context of endoscopic transsphenoidal pituitary adenoma resection using an international, expert-driven consensus process. Building on our previously published operative workflow for this procedure [1], the current Delphi analysis shifts the focus from task sequencing and how surgery is performed to phase-specific visual confirmation criteria and how readiness to progress is determined. It introduces a novel framework aimed at supporting safe intraoperative decision-making and enhancing standardisation across surgical practice.

Using a modified Delphi methodology, we identified a core set of phase-specific essential and desirable visual cues that define safe progression between major surgical phases (i.e., naso-sphenoid, sellar, closure). Our proposed CVPP framework adapts principles from surgical safety, systems engineering, and workflow science to define what surgeons must visualise before advancing to the next stage of surgery [22, 23, 28]. The agreed CVPPs can be used for education (e.g., operative video annotation), surgical skills assessment, clinical auditing, and the development of surgical safety checklists, models, and simulators [21, 29–32].

This work is a product of an international, multicentre consensus, capturing variations in surgical practice and training backgrounds. Given this breadth of representation, achieving a structured framework and maintaining consistent terminology proved challenging and required several iterative refinements across multiple Delphi rounds. Early discussions highlighted variability in exposure standards and anatomical emphasis among experts, depending on the operative contexts (e.g., tumour size, invasiveness), which prompted the creation of separate CVPPs for macro- and microadenoma cases. Additional complexity arose because the study introduced a novel concept that had not been previously defined in the literature. Iterative refinement across Delphi rounds ensured experts shared a consistent understanding of the terminology and definitions, using examples to resolve interpretative discrepancies and accommodate the full spectrum of operative practice. This ultimately produced a unified framework that is both methodologically consistent and flexible enough to reflect real-world variability in surgical decision-making within a shared safety structure.

The validation of the CVPP framework confirmed high overall concordance between expert judgement and the predefined reference classifications, supporting its face validity and clinical reliability. While lower levels of agreement were observed for intentionally incomplete or “unsafe” views, qualitative feedback suggested that these discrepancies in the decision to proceed arose from a contextual misinterpretation of the short video clips (i.e., a lack of procedural context) rather than disagreement with the visual cues themselves. This confirms that CVPPs reliably capture critical visual information that surgeons use, often intuitively, to judge intraoperative readiness to proceed. It is important to note that the present study was designed as a face-validity assessment of the CVPP framework rather than an educational intervention and future work is needed to evaluate whether the structured pre- and post-educational exposure improves overall performance.

### Findings in the context of existing literature

The concept of visually confirming safety before procedural progression is not new to surgery but has rarely been formalised beyond specific specialities. The most established example is Strasberg’s “Critical View of Safety” in LC [9, 11, 13, 16], which significantly reduced iatrogenic bile duct injury through direct visual confirmation of key anatomical landmarks (hepatocystic triangle, cystic plate, cystic duct and artery) before transection of the cystic duct and artery [10, 33–35]. Subsequent studies have validated the utility of CVS as both a teaching and safety tool, demonstrating how structured visual verification at critical transition points can improve outcomes, enable standardised analysis of intraoperative performance, and guide the development of intelligent systems [20, 35–38]. Audit-based initiatives using intraoperative photo and video documentation of CVS have shown that satisfactory visual confirmation strongly correlates with lower rates of postoperative complications, establishing CVS adherence as a measurable quality indicator in institutional and national audits [35, 38]. In surgical education, structured curricula have been shown to improve surgeons’ attainment of CVS [36], while studies also highlight that self-assessment often overestimates performance [38], highlighting the need for objective feedback and standardised assessment frameworks. Building on these efforts, Rios et al. recently introduced the Cholec80-CVS – an open dataset providing expert-annotated operative footage for the development and benchmarking of CV models capable of automatically detecting anatomical landmarks, validating CVS achievement, and flagging unsafe dissection zones in real time [20]. These developments demonstrate how the CVS framework has evolved from a visual safety principle to a foundation for quality improvement, training, and AI-driven surgical guidance – all directly translatable to the proposed CVPP framework. However, while CVPPs draw inspiration from the CVS, the two frameworks differ fundamentally in scope and intent. The CVS principle focuses on a single anatomical juncture with a clearly defined adverse outcome, therefore its strength lies in the simplicity and specificity of the intervention. The CVPP framework expands on this by embedding visual confirmation into every major surgical phase transition, establishing a continuous system of structured checkpoints that collectively define safe operative progression within a complex, context-dependent procedure. As a result, a direct one-to-one translation of the CVS concept is neither feasible nor intended. The CVPP framework deliberately operates at a higher level of abstraction, defining minimum visual criteria that indicate readiness to progress between surgical phases rather than attempting to encode the full nuance of operative technique or expert judgement. This abstraction is intentional and enables complex surgical workflows to be structured around predefined safety checkpoints without constraining case-specific strategy.

Beyond its surgical roots, the CVPP framework draws inspiration from engineering risk management principles. In systems engineering, the phase-gate (“waterfall”) process is a technique in which a project is divided into distinct stages (“phases”), separated by decision points (“gates”) [22, 23]. This provides a structured mechanism for assessing readiness and mitigating risk by ensuring that each developmental stage is advanced only once specific, objectively verifiable criteria have been met [22, 23, 39, 40]. Applying this philosophy to the operative environment, the CVPP framework introduces a similar mechanism of safety gating within surgery, integrating a proactive form of hazard control into the operative workflow – one that can be standardised, auditable, and amenable to future digital translation. Engineering-derived frameworks are increasingly recognised in healthcare for their ability to standardise complex processes, reduce cognitive load, and minimise error propagation – all of which align with the principles of modern surgical safety systems [41–44]. This cross-disciplinary translation situates the CVPP framework within a growing movement towards structured, data-driven, cognitively intuitive surgery. Efforts to standardise operative workflows and develop machine-readable frameworks have been increasingly more common across various surgical fields – from minimally invasive general surgery to robotic procedures [45–49]. In this context, CVPPs represent a natural progression from our previously published workflow, translating implicit decision-making into explicit and quantifiable data.

Although applied to pituitary surgery in this study, the CVPP model is inherently procedure-agnostic. Its principles (i.e., structured progression, predefined safety criteria, and visual confirmation) are universally applicable across surgical fields where phase-based transitions occur (e.g., robotic-assisted surgery). The framework’s modularity allows local or speciality-specific visual criteria to be defined, validated, and incorporated into training, quality improvement systems and safety checklists. As such, CVPPs offer a scalable foundation for intraoperative safety validation and future automation across diverse operative settings.

Finally, a defining feature of the CVPP framework is its machine-readable structure. By formalising phase completion as a finite set of visually verifiable cues, CVPPs provide ground truth suitable for training CV models in phase recognition, anatomical landmark detection, and automated safety validation. This positions CVPPs as a bridge between clinical reasoning and machine-readable safety benchmarks, shifting surgical AI models from purely descriptive workflow and anatomy recognition toward explicit safety verification, expanding existing workflow and anatomy-recognition efforts in endoscopic transsphenoidal surgery [50–52].

### Clinical implications

CVPPs are intentionally designed to raise the floor, not the roof of surgical performance. Their primary aim is, therefore, to define the minimum visual criteria a surgeon would reasonably expect to satisfy before phase progression, rather than to modify expert surgical technique. By making these safety-critical visual objectives explicit, reproducible, and assessable, CVPPs may provide a structured framework for use in training, audit, and structured assessment purposes. Clinically, the CVPP framework provides a reproducible tool for intraoperative verification and surgical audit with the potential to standardise intraoperative decision-making (e.g., akin to WHO Surgical Safety Checklist [28]). From a safety perspective, embedding visual cues into intraoperative checklists could reinforce visual verification as a formalised step before proceeding. Educationally, CVPPs transform tacit visual information into teachable and assessable benchmarks for surgical trainees, enabling structured feedback and objective assessment of competency-based progression. Evidence from CVS studies [36, 53] suggests that integrating CVPPs into surgical curricula or video-based learning may enhance recognition of the anatomical and procedural hallmarks underpinning safe phase completion, bridging the gap between observation and independent practice. The local validation study intentionally included surgeons at various stages of training and independent practice, including fellows and residents, who stand to benefit most from structured visual frameworks.

Beyond immediate clinical use, the annotated nature of CVPPs provides a foundation for future integration of AI and CV tools capable of automatically recognising critical anatomical views and phase transitions. As demonstrated in recent work on automated CVS detections, models trained on labelled operative footage can reliably detect safety-critical views in real time [18, 19, 21]. Within endoscopic transsphenoidal surgery specifically, recent advances in step segmentation and anatomical recognition provide the technical foundation for such systems [54, 55]. CVPP-labelled datasets could supply the phase-completion criteria missing from current models, linking visual understanding to actionable intraoperative decision support.

### Strengths & limitations

A major strength of this study was its international scope, which captured practices from multiple institutions and diverse surgical backgrounds. The expert panel, drawn from our established international research network, brought recognised expertise in pituitary and skull base surgery, ensuring that the framework reflected a broad range of operative practices and anatomical interpretations. While the panel spanned Europe and North America, the absence of low- and middle-income country representation means that our results may not fully reflect global practices. Furthermore, the framework was intentionally designed to capture the minimum, safety-critical visual cues applicable across a wide range of cases – an approach that prioritises reproducibility and consistency, but does not attempt to capture case-specific anatomical variation (e.g., sphenoid pneumatisation patterns, skull base defects) or patient-specific surgical objectives. Consequently, experienced surgeons are unlikely to derive additional operative guidance from these cues – a limitation that reflects the intended scope of the framework.

Beyond this, some methodological limitations should also be acknowledged. First, the validation study was conducted within a single tertiary centre and involved a small cohort, which may limit the generalisability of our findings. Second, while the CVPPs formalise the visual readiness for phase progression, they do not account for dynamic elements of intraoperative decision-making (e.g., instrument handling, navigation data). Third, the nasal and sphenoid phases were combined into a single naso-sphenoid phase to facilitate future digital annotation. While this improved phase stability for data analysis, it may limit the granularity of workflow characterisations for studies focusing on the early exposure phase. Fourth, regarding the validation study, the use of short, decontextualised video clips may have contributed to isolated instances of interpretive variability. In addition, formal inter-rater reliability statistics (e.g., Cohen’s κ) were not calculated, as the sample size and purposive clip selection could render such metrics unstable and potentially misleading.

Ultimately, this framework provides a foundation for a universal phase-gate approach to intraoperative decision-making. Future work should focus on expanding the validation study across multiple centres, including pre- and post-educational intervention designs, and prospective integration of CVPPs into intraoperative workflows to assess their impact on surgical outcomes, learning curves and efficiency. As annotated datasets expand, CVPPs can serve as structured ground truth for CV models capable of real-time phase recognition, automated safety validation, and performance benchmarking.

## Conclusion

Through an international expert consensus process, this study establishes the first framework of “Critical Views for Phase Progression” and applies it to endoscopic transsphenoidal pituitary surgery. The CVPP framework bridges engineering-derived phase-gate principles with surgical safety, introducing a structured and verifiable approach to intraoperative decision-making and offering a scalable foundation for training, audit, and future digital integration.

## Data Availability

Available upon reasonable request.

## Appendix A: example intraoperative images illustrating a potential view at the end of each surgical phase for macro- and microadenomas

(A) Potential intraoperative view at the end of naso-sphenoid phase - macroadenoma

(B) Potential intraoperative view at the end of naso-sphenoid phase - microadenoma

(C) Potential intraoperative view at the end of sphenoid phase - macroadenoma

(D) Potential intraoperative view at the end of sphenoid phase - microadenoma

(E) Potential intraoperative views at the end of closure phase

## Appendix B: guidance questions to experts during each consensus round

### Phase 1: Naso-sphenoid phase

*Q1*: When establishing critical view for safe progression at the end of naso-sphenoid phase, how would you classify the importance of visualising the following landmarks for safe progression to the sellar phase?

*Q2*: Are there any additional landmarks you believe should be considered **essential** to visualise at the end of the naso-sphenoid phase?

*Q3*: Are there any additional landmarks you believe should be considered **desirable** to visualise at the end of the naso-sphenoid phase?

### Phase 2: Sellar phase

*Q4*: When establishing critical view for safe progression at the end of sellar phase, how would you classify the importance of visualising the following landmarks for safe progression to the closure phase?

*Q5*: Are there any additional landmarks you believe should be considered **essential** to visualise at the end of the sellar phase?

*Q6*: Are there any additional landmarks you believe should be considered **desirable** to visualise at the end of the sellar phase?

### Phase 3: Closure phase

*Q7*: When establishing critical view for safe progression at the end of closure phase, how would you classify the importance of visualising the following landmarks?

*Q8*: Are there any additional landmarks you believe should be considered **essential** to visualise at the end of the closure phase?

*Q9*: Are there any additional landmarks you believe should be considered **desirable** to visualise at the end of the closure phase?

### General questions

*Q10*: Do you have any suggestions for improving the identification or visualization of landmarks during surgery?

## Abbreviations

AI: artificial intelligence
CSF: cerebrospinal fluid
CV: computer vision
CVPP: critical view for phase progression
CVS: critical view of safety
LC: laparoscopic cholecystectomy
ONP: optic nerve prominences
UK: United Kingdom
USA: United States of America
